# Identifying effective behavioural components of Intervention and Comparison group support provided in SMOKing cEssation (IC-SMOKE) interventions: a systematic review protocol

**DOI:** 10.1186/s13643-016-0253-1

**Published:** 2016-05-04

**Authors:** Marijn de Bruin, Wolfgang Viechtbauer, Maarten C. Eisma, Jamie Hartmann-Boyce, Robert West, Eleanor Bull, Susan Michie, Marie Johnston

**Affiliations:** Aberdeen Health Psychology Group, Institute of Applied Health Sciences, University of Aberdeen, Aberdeen, Scotland; Department of Psychiatry and Neuropsychology, School for Mental Health and Neuroscience, Maastricht University, Maastricht, Netherlands; Nuffield Department of Primary Care Health Sciences, University of Oxford, Oxford, England; Health Behaviour Research Centre, Department of Epidemiology and Public Health, University College London, London, England; NHS Grampian Public Health Directorate, Aberdeen, Scotland; Research Department of Clinical, Educational and Health Psychology, University College London, London, England

**Keywords:** Smoking cessation, Systematic review, Meta-analysis, Meta-regression, Behaviour change technique, Intervention, Comparison group, Control group, Randomized controlled trial, Tobacco

## Abstract

**Background:**

Systematic reviews of behaviour change interventions for smoking cessation vary in scope, quality, and applicability. The current review aims to generate more accurate and useful findings by (1) a detailed analysis of intervention elements that change behaviour (i.e. behaviour change techniques (BCTs)) and potential moderators of behaviour change (i.e. other intervention and sample characteristics) and (2) assessing and controlling for variability in support provided to comparison groups in smoking cessation trials.

**Methods:**

A systematic review will be conducted of randomized controlled trials of behaviour change interventions for smoking cessation in adults (with or without pharmacological support), with a minimum follow-up of 6 months, published after 1995. Eligible articles will be identified through the Cochrane Tobacco Addiction Group Specialized Register. Study authors will be asked for detailed descriptions of smoking cessation support provided to intervention and comparison groups. All data will be independently coded by two researchers. The BCT taxonomy v1 (tailored to smoking cessation interventions) and template for intervention description and replication criteria will be used to code intervention characteristics. Data collection will further include sample and trial characteristics and outcome data (smoking cessation rates). Multilevel mixed-effects meta-regression models will be used to examine which BCTs and/or BCT clusters delivered to intervention and comparison groups explain smoking cessation rates in treatment arms (and effect sizes) and what key moderators of behaviour change are. Predicted effect sizes of each intervention will be computed assuming all interventions are compared against comparison groups receiving the same levels of behavioural support (i.e. low, medium, and high levels). Multi-disciplinary advisory board members (policymakers, health care providers, and (ex-)smokers) will provide strategic input throughout the project to ensure the review’s applicability to policy and practice.

**Discussion:**

By capturing BCTs in intervention and comparison groups, this systematic review will provide more accurate estimates of the effectiveness of smoking cessation interventions, the most promising BCTs and/or BCT clusters associated with smoking cessation rates in intervention and comparison arms, and important moderators of behaviour change. The results could set new standards for conducting meta-analyses of behaviour change interventions and improve research, service delivery, and training in the area of smoking cessation.

**Systematic review registration:**

PROSPERO CRD42015025251

**Electronic supplementary material:**

The online version of this article (doi:10.1186/s13643-016-0253-1) contains supplementary material, which is available to authorized users.

## Background

Smoking is an important cause of preventable disease and disability, which leads to a reduced quality of life and life expectancy, an increase in health care burden, and loss of productivity for society [1, 2]. Numerous behaviour change interventions for smoking cessation have been developed and evaluated in randomized controlled trials (RCTs). These trials have been synthesized in multiple systematic reviews including meta-analyses, which enable researchers, practitioners, and policymakers to draw conclusions based on the totality of available evidence [3]. The strength of these meta-analyses lies in their ability to identify the most effective interventions and to identify intervention characteristics associated with greater intervention effectiveness across settings and populations.

Recently, there have been two important methodological developments in the field of systematic reviewing and meta-analysis of behaviour change interventions that are important to these outcomes but have not yet been applied to smoking cessation trials. First, a more advanced system for reliably specifying the content of behaviour change interventions has been developed, resulting in more sophisticated and informative meta-analyses of the active content of behaviour change interventions [4–7]. The second innovation builds on the first, but represents a step-change in how we interpret and compare results from behaviour change interventions in research syntheses. Comparison groups, like intervention groups, often receive behavioural support that varies considerably in content and effectiveness between trials. Taking that into account in meta-analyses can significantly alter conclusions about the ‘true’ effect sizes of interventions [5, 8, 9]. To present unbiased and accurate recommendations about what interventions work and under what conditions, it is vital to take variability in comparison group contact into account [5, 8–11].

Evidence is accumulating that these innovations can substantially advance the quality and usefulness of meta-analyses by more accurately estimating true intervention effects and the most effective elements of intervention and comparison group treatment [5, 11–14]. The aim of the current study is to adapt these innovative methods and apply them in a comprehensive, state-of-the-art systematic review and meta-analysis of smoking cessation interventions. This work could be a major step forward in increasing the effectiveness of smoking cessation RCTs and their applicability to practice.

### Coding active content of interventions: behaviour change techniques

Previous reviews have typically focussed on segments of the smoking cessation literature, based on, for example, ‘by whom’ interventions were delivered (e.g. a physician, pharmacist, nurse) [15–17], ‘how’ they were delivered (e.g. face-to-face counselling, internet, telephone support) [18–20], or ‘where’ they were delivered (e.g. workplace, health, or community settings) [21, 22]. This approach is limited in that it is unable to identify if these intervention characteristics are important moderators of intervention effectiveness. Moreover, within these reviews, interventions are often grouped based on fairly broad and heterogeneous characteristics that do not describe intervention content in sufficient depth. For example, stop smoking advice from physicians is often termed ‘brief advice’ but may include presenting a leaflet, individual counselling of varying durations, referral to smoking cessation programmes, or biofeedback using a carbon monoxide monitor [15]. Given these differences in content and scope, it is unsurprising that systematic reviews have observed significant variability in effectiveness of ‘brief advice’ intervention (smoking cessation rates of 0–20 % [15]). Currently, systematic reviews only rarely focus on the active content of behaviour change, called behaviour change techniques (BCTs), as a possible explanation for this variability [4, 6, 7, 23].

BCTs constitute the smallest active ingredients of interventions capable of inducing change in behaviour in appropriate circumstances [7]. For example, offering money or vouchers for each month without smoking would be coded as ‘material incentive’, and setting a quit date as ‘goal setting’. Achieving reliable coding of active content of behaviour change interventions is complex; smoking cessation interventions may employ up to 56 BCTs [24]. An extensive research programme has developed taxonomies of BCTs (with precise labels and definitions) which achieve good reliability in coding BCTs from behaviour change intervention descriptions [25]. This work has advanced research syntheses in fields such as medication adherence and weight loss interventions, by describing interventions in a replicable format and using meta-regression to explore *why* interventions work (e.g. [5, 12–14, 26]). This work includes a taxonomy for smoking cessation interventions and studies examining how these can be reliably applied [6, 25, 27–30]. We now propose to conduct detailed content-coding of behaviour change interventions for smoking cessation using the behaviour change technique taxonomy version 1 (BCTTv1) [7] and incorporate this in state-of-the-art meta-analyses in order to identify what works best for whom under which circumstances. In addition to coding intervention content (i.e. BCTs), we will use the template for intervention description and replication (TIDieR) to code other key intervention characteristics that may serve as moderators of BCT effectiveness, such as the setting, route of delivery, and discipline and training of the person delivering the intervention [31].

### Coding active content of comparison groups

The second innovation applied in this project is to apply the same rationale to comparison groups as to intervention groups in trials of smoking cessation interventions (i.e. to code BCTs and moderators of BCT effectiveness). Comparison groups often also receive behavioural support that may vary considerably in content and effectiveness. Meta-analyses that do not account for this variability may produce inaccurate estimates of intervention effects and their active content [9]. We recently examined the content, variability, and effectiveness of BCTs provided to comparison groups in behaviour change interventions for the first time. Focusing on RCTs of counselling programmes to promote medication adherence, a reliable index was developed for quantifying the active content (weighting the number and type of BCTs delivered) of what authors described as ‘usual/standard care’ provided to comparison groups [5, 8]. This active content score varied considerably between ‘treatment-as-usual comparison groups’, was the main predictor of treatment success rates in comparison groups [8], and in turn had an impact on the effect sizes of interventions tested (i.e. effect sizes were smaller in ‘treatment-as-usual settings’ where many BCTs, often similar to those employed in interventions, were already being delivered). This new method of assessing the active content of comparison group support has made it possible to obtain more valid estimates of the effectiveness of the interventions tested (i.e. by adjusting intervention effect estimates for the variability in support provided to comparison groups), and the intervention ingredients responsible for these effects [5, 8].

These procedures have not yet been applied in the field of smoking cessation, yet there are strong indications that comparison group variability is also relevant for evidence syntheses of smoking cessation trials. First, current reviews report substantial variability in the content and scope of support provided to comparison groups (e.g. no intervention, treatment-as-usual, self-help materials, information only). Cochrane reviews investigating comparison group characteristics define broad subgroups based on intensity (e.g. number of sessions) or type (e.g. treatment-as-usual versus self-help materials) rather than by their active content (i.e. BCTs). Second, primary care smoking cessation support outside the context of research trials shows substantial variability in treatment-as-usual between practices/practitioners (e.g. [32, 33]). Third, smoking cessation rates in comparison groups vary considerably within and between comparison group types; in fact, comparison group cessation rates are often comparable to those observed in the intervention groups (e.g. 0–30 % for intervention versus 0–28 % for comparison groups) [18]. These data strongly suggest that when interpreting and comparing effects of smoking cessation interventions, the active content of support delivered to the comparison groups should be taken into account; this might explain the observed variability of success rates in comparison groups and, consequently, in intervention effect sizes [5, 7–10].

### Objectives

We propose to build on the most recent Cochrane reviews of behaviour change interventions for smoking cessation and introduce these methodological innovations in a comprehensive, rigorous systematic review.

The objectives are:

1. To examine whether variability in effectiveness of smoking cessation interventions evaluated in RCTs can be better explained through improved characterization of the active content of:
  - The behaviour change interventions
  - The behavioural support provided to comparison groups in these trials
2. To make recommendations to practitioners and policymakers about the most effective ingredients of behaviour change interventions for smoking cessation, their impact, and the conditions under which they are most likely to be effective

Research questions are:

1. Which BCTs and/or BCT clusters provided to intervention and comparison groups explain heterogeneity in smoking cessation rates in intervention and comparison groups in RCTs on smoking cessation interventions and in effect sizes?
2. What are the key moderators of effectiveness of BCTs in smoking cessation interventions?
3. To what extent are intervention effect sizes influenced by differences in the behavioural support provided to comparison groups in trials?

## Methods

### Reporting standards

The review is registered with PROSPERO (international prospective register of systematic reviews) at the National Institute for Health Research and Centre for Reviews and Dissemination (CRD) at the University of York (registration number CRD42015025251) and will be reported in accordance with the PRISMA statement ([34], Additional file 1).

### Overview of literature search

The Cochrane Tobacco Addiction Review Group Specialized Register (CTAGSR) (via the Cochrane Register of Studies Online (CRSO) will be searched for studies published from 1996 onwards (see http://onlinelibrary.wiley.com/o/cochrane/clabout/articles/TOBACCO/frame.html). This register is developed through continued and regular electronic searching of MEDLINE, EMBASE, and PsycINFO, together with hand searching of specialist journals, conference proceedings, and reference lists of previous trials and overviews. The CTAGSR is indexed on whether or not studies are included in existing Cochrane reviews. The current reviews’ inclusion criteria for study design, population, and outcome are based on those used by the Cochrane tobacco addiction group.

### Search and screening process

Inclusion criteria

- RCTs with a minimum follow-up period of 6 months
- Interventions directed at adult smokers
- Trials describing (1) behaviour change interventions with or without pharmacotherapy compared to (a) different behaviour change interventions with or without pharmacotherapy, (b) treatment-as-usual with or without pharmacotherapy, (c) pharmacotherapy alone, or (d) no treatment.
- Smoking cessation rates (primary outcome) reported at least 6 months after the start of the intervention
- Trials published in peer-reviewed journals from 1996 onward
- Trials published in English

Trials describing behaviour change interventions, which we have defined as ‘(a set of) materials and/or activities designed to change behaviour through social and psychological processes’ [35, 36], will be included in our review. Only trials published in the last 20 years will be considered for inclusion for two reasons. First, more recent trials will be more relevant to current practice. Second, it was judged to be unlikely we could retrieve accurate information about intervention and comparison groups for trials published longer ago. Only trials published in English will be included, because no resources are available for the translation of intervention and comparison group protocols/materials, or the trial paper.

Searching and screening of the CTAGSR will be enhanced through use of existing Cochrane reviews using the following procedures. First, all potentially relevant references included in CTAGSR published from 1996 onward will be obtained (date initial search: 1-11-2015; 5989 references). Next, the study exclusion lists of published Cochrane systematic reviews will be used to remove studies based on shared inclusion/exclusion criteria (e.g. follow-up shorter than 6 months, not an RCT). Third, all remaining studies (titles and abstracts) will be screened independently by two reviewers (exclude at title/abstract stage or proceed to full-text screening). Fourth, the full-text published articles of references selected at title/abstract stage will be assessed. Throughout this process data will be managed using Covidence, Endnote, and MS Excel.

Since there is a substantial number of papers currently included in the full-text screening phase (about 1500), these papers will be divided into two sets: one set that is double-screened and a set that is screened by one person. To minimize the risk of eligible trials being excluded, and ineligible trials being included, the following strategy will be used:

1. Paper titles will be organized using the standard options in Covidence, the Cochrane systematic review tool, and the first 200 papers will be selected to be double-screened (eligible or not eligible) by the three screeners working on this study (each screening approximately 140 papers). Disagreements will be discussed, and if not resolved, the third screener will be consulted. The process includes reflection on biases in the selection of papers leading to disagreements, in particular regarding the exclusion of eligible trials. Agreement will be considered satisfactory if prevalence-adjusted bias-adjusted kappa (PABAK) is >.80 (indicating excellent inter-rater reliability [37]). If inter-screener agreement is unsatisfactory, the same procedure will be repeated until satisfactory agreement is achieved. If/when agreement is satisfactory, three screeners will start to single-screen one third of all the references. The screeners were instructed that in case of uncertainty, they should include the paper with a comment stating ‘Note sure’ to minimize the risk of excluding eligible trials.
2. After the screeners have examined 50 % of their papers, each will examine 30 randomly selected papers (already screened) from one of the other screeners, allowing for a repeated comparison of consensus on another 90 papers. The same procedure will be applied for checking reliability as in step 1.
3. All the papers included by one screener (i.e. eligible) will be assigned to at least one other screener in the data extraction phase, who will then confirm if study inclusion was justified. Thus, all included papers will have been included by two screeners, minimizing the risk of including ineligible trials.

### Data collection

#### Acquiring information about intervention and comparison groups in included studies

The content of interventions, and especially of comparison group support, is usually described in insufficient detail in published articles to allow coding of BCTs. Article authors will therefore be contacted and asked to provide additional materials that describe the intervention in more detail. This can be the full intervention manuals, materials (e.g. website content, folders), or materials to train professionals in the delivery of the intervention. We will first contact authors by email (including reminders) and follow up by telephone and/or fax if an author does not respond. If the first author cannot be contacted, the second and last authors will be approached.

Authors will also be asked for details regarding comparison group support, which may consist of (1) smoking cessation support introduced by researchers (e.g. self-help materials)—for these, we will request all relevant manuals/materials, similar to the interventions—and (2) treatment-as-usual (TAU) smoking cessation support delivered to participants by their health care providers during the study. As TAU delivered in trials is typically not formally documented, a TAU checklist will be developed and sent to authors to fill out. This checklist contains a comprehensive list of TAU activities to support smoking cessation identified through (1) BCT coding four international stop smoking treatment manuals [38–41]; (2) input from an advisory board of (ex-)smokers, smoking cessation professionals, and policymakers; (3) expertise in the team on smoking cessation services, developed through previous studies (including recording and BCT coding English smoking cessation service sessions [42]), and attending smoking cessation sessions; and (4) smoking cessation example activities described in previous (smoking cessation) BCT taxonomies [6, 7, 42]. Only activities identified as the application of a BCT will be included as an item in the checklist.

Study authors will be asked to indicate which activities in the checklist were part of TAU. Activities that are considered to be part of TAU are those that have been ‘routinely delivered to the majority of comparison group participants during the trial’. This approach to retrospectively obtaining TAU information on comparison groups proved feasible in previous reviews: data was obtained from 67 % of the studies conducted in a clinical setting and yielded data that was internally consistent (Cronbach alpha >.90) with substantial predictive validity (i.e. variability in the scores on this checklist predicted substantial variability in objective comparison group outcomes and intervention effects) [5, 8]. Authors will also be asked whether only the comparison group or also the intervention group was exposed to TAU.

#### Data extraction

The following information will be coded independently by two reviewers:

1. Sample characteristics and inclusion/exclusion criteria.
2. Trial characteristics such as risk of bias (using the Cochrane risk of bias tool [3], complemented with elements described in the RATIONALE table [43]) and other potential effect modifiers.
3. Smoking cessation rates (objective and subjective measures) at each time point reported. Secondary outcomes are mortality and morbidity. Both available case and intention-to-treat data will be collected.
4. The *active content* (BCTs delivered to promote smoking cessation) of the behavioural support delivered to both intervention and comparison groups will be coded from the treatment manuals/materials and the TAU checklist, using the BCTTv1 ([7]), which will be adapted to the smoking cessation context, by adding smoking cessation examples of BCTs (focused on quitting smoking, abstaining from smoking, and medication adherence), introducing new BCTs if identified in the coding process, and removing BCTs irrelevant to smoking cessation interventions. It will also be coded if pharmacotherapy is used and what type of smoking cessation medication is used.
5. Intervention and comparison group characteristics that serve as *potential moderators* of BCT effectiveness, such as the professional delivering the intervention, setting, or the medium; duration of the intervention; and tailoring and repetition of BCTs [5, 8, 31, 36].
6. Fidelity measures for intervention implementation.

Differences in data extraction for these variables will be discussed by both coders until consensus is reached. If consensus cannot be reached, the opinion of a third coder and, if necessary, other members of the research team will be consulted to determine the final decision. For each BCT and coded moderator of smoking cessation support, inter-rater reliability will be examined using adjusted Kappa statistics [37, 44]. Items with suboptimal reliability (i.e. <.7) will be discussed with a third coder and, if necessary, the wider research team. Discrepancies that are not resolved with high confidence (no agreement after discussion) and items with low endorsement frequencies (present in <5 % of the trials) will be noted but not used in further analyses. Similar procedures will be used for the other elements, such as risk of bias and fidelity measures.

### Statistical analyses

Only eligible studies (i.e. that fit the inclusion criteria) for which we are able to extract the primary outcome of interest (i.e. cessation rate) for at least one study arm and one time point and for which we are able to code the intervention content will be included in the main analyses.

#### Step 1: identifying the active components of smoking cessation support provided to intervention and comparison groups and their impact on effect sizes

BCTs may be examined individually, but as far as possible they will be analysed as clusters based on (i) behavioural theory (e.g. [10]), (ii) cluster analyses (e.g. [45]), and (iii) a sum score of all BCTs delivered, including a weighting for delivery factors associated with outcomes in step 2 (e.g. level of tailoring and repetition of BCTs, e.g. 3). We will first examine which (clusters of) BCTs delivered to intervention groups, and which (clusters of) BCTs provided to comparison groups explain heterogeneity in smoking cessation rates in the respective arms. For this, the cessation rates of both arms will be used as the outcomes of interest and analysed by using an arm-based multilevel mixed-effects meta-regression model that allows the true rates of the intervention and comparison group from the same study to be correlated [46, 47]. The effects of (clusters of) BCTs on smoking cessation rates will be allowed to differ between intervention and comparison groups by including a dummy variable indicating the group and its interaction with the BCT cluster/moderator score in the model. Hence, this model not only provides the information that a conventional mixed-effects meta-regression analysis based on success rate differences (i.e. the effect sizes) would offer but also (a) allows the inclusion of one-arm studies (e.g. when BCT clusters can only be coded for the intervention or control arm of a study) [47–49], (b) indicates which (clusters of) control and intervention BCTs are related to cessation rates on an absolute scale, and (c) can thus reveal which effective (clusters of) *intervention BCTs* account for an *increase* in effect sizes and what effective (clusters of) *comparison group BCTs* account for a *decrease* in the effect sizes.

Time points used will be (at least) immediate post-intervention (to identify BCT clusters related to initial smoking cessation) and at the last follow-up (to identify BCT clusters related to cessation maintenance), while controlling for differences in follow-up duration between studies. We will also explore the use of appropriate multivariate models to meta-analyse the entirety of the available evidence at once (i.e. multiple time points) [50, 51].

Additionally, using an extension of the Egger regression test to an arm-based model, the presence of small-study effects (which may be indicative of potential publication bias) will be examined by including the inverse square root sample size and its interaction with the intervention/comparison group dummy variable as predictors in the meta-regression model [52]. A significant interaction would indicate that the size of the differences in cessation rates between the intervention and comparison groups is related to the standard errors by which the rate differences are measured (and hence the statistical significance of the cessation rate differences within studies).

#### Step 2: identifying moderators of BCT effectiveness

Key moderators of effective (clusters of) BCTs are intervention attributes described in TIDieR, mainly setting, provider, and route of delivery, and sample characteristics such as socio-economic status or intention to quit smoking at baseline. All analyses will be controlled for potential covariates such as study design (e.g. risk of bias), sample characteristics (e.g. age, gender, health status of participants), pharmacotherapy, and BCT co-occurrence and take into account issues with multiple-testing [10].

#### Step 3: estimating intervention effect sizes adjusted for comparison group variability

If the smoking cessation support provided to comparison groups varies between trials and impacts effect sizes, we will compute an active content index (weighing (sets of) effective comparison group BCTs) and use the model to compute the predicted effect size of each intervention assuming it had been compared against similar comparison groups (i.e. under low, medium, or high amounts of behavioural support) [3]. This model should provide a more even-handed comparison of the effectiveness of the various interventions than the currently published evidence.

### Advisory board

Multi-disciplinary advisory board members (policymakers, health care providers, and (ex-) smokers) will provide essential strategic input throughout the project to ensure the review’s usefulness for policymakers, practitioners, and the public (smokers). The advisory board will meet with members of the research team three times during the project to provide strategic input at specific stages of the project: during (1) initial research design, (2) data analysis and interpretation of results, and (3) research dissemination planning. The first advisory board meetings have already been held. Important contributions of advisory board members have been an improvement of the selection of control variables and potential moderators of BCT effectiveness and advising on the selection and phrasing of items for the TAU activity list.

### Data sharing

Data used in scientific manuscripts will be shared online upon manuscript acceptance. Unused data will be published online no later than 12 months after project completion (to permit the applicants to generate additional scientific output).

### Dissemination of findings

The results of this project will be published in international peer-reviewed journals and presented at (inter)national scientific conferences. A study website will be developed to disseminate the results to researchers, policymakers, practitioners, and the general public. Our advisory board will provide recommendations and feedback regarding website design and content, which will be tailored for specific audiences. The policymakers’ section will contain a summary of the main outcomes (including an evidence strength grading table) and study implications for policy and practice. The researcher section will contain all data, measures, computer programme syntaxes for statistical analyses, outputs, and published manuscripts. In case of evidence of sufficient strength, the practitioners’ section will contain a self-assessment tool for the active content of the smoking cessation support currently provided and will provide feedback in the form of recommendations for increasing the effectiveness of their practice. Video examples of how to deliver effective (clusters of) BCTs will support implementation of these recommendations. The general public section will provide a summary of the project outcomes and key recommendations, such as effective self-help strategies, and referral to local smoking cessation services. Lastly, the data and results (which will be assessed using GRADE criteria [3]) will be sent to the appropriate bodies within and outside of the UK for consideration in their guidelines for smoking cessation support (e.g. National Institute for Health and Care Excellence, UK; the UK National Health Service Stop Smoking Services: Monitoring and Guidance; Tobacco Use and Dependence Guideline Panel from the US Department of Health and Human Services).

## Discussion

By capturing BCTs in both intervention and comparison groups, this systematic review advances current methods and evidence and should provide more accurate estimates of the true effectiveness of smoking cessation interventions taking into account the diversity of comparison groups in reported trials. It will identify the most promising (clusters of) BCTs associated with smoking cessation rates and pinpoint important moderators of intervention effectiveness. The results will yield valuable data for policymakers and smoking cessation service providers, offering guidance about effective support and about training needed for delivery of effective services. The methods developed in this project could also set new standards for conducting systematic reviews and meta-analyses of behaviour change interventions and, more widely, in reviews of health care interventions.

A number of challenges may arise while conducting this systematic review, some of which warrant brief mention here. In order to perform the review, we need additional information about intervention and comparison group support from a substantial number of authors of smoking cessation RCTs. While some previous studies have yielded a good author response rate [8, 53], it remains to be established whether such response rates can be achieved in this context. Although there are many eligible trials, substantial difficulties in receiving additional materials from trial authors may limit the power of the proposed analyses. In this case, less elaborate analyses (i.e. fewer BCT predictors and moderators), which require smaller sample sizes, will be conducted. Similarly, comparison group data collected from authors using the TAU checklist may be less accurate for older than for recent studies. This issue can be explored by examining whether the internal consistency of the TAU checklist depends on publication year and by examining the frequency of the number of ‘don’t know’ responses to checklist items across publication years. If a potential issue with reporting accuracy is identified, we will include an ‘accuracy’ variable in the main analyses (e.g. the number of ‘don’t know’ responses in the TAU checklist) to see if this affects the results. Another challenge may be that a larger number of eligible trials is available than can be coded using available resources (e.g. >200–250 trials). In this case we will include those trials with a combination of high methodological quality (e.g. trials with objectively verified smoking cessation rates) and good information about intervention and comparison groups and primary outcomes.

## Conclusion

In summary, this systematic review has the potential to identify what behavioural components of smoking cessation interventions work best for whom under which circumstances. The findings can inform future public health practice and policy aimed at smoking cessation. Additionally, the innovations applied in this study could set new standards for meta-analyses, both in the field of smoking cessation and in systematic reviews of health care interventions more generally.

### Ethics approval and consent to participate

Not applicable.

### Consent for publication

Not applicable.

## Additional file

Additional file 1: Prisma checklist. (DOCX 41 kb)

## Abbreviations

BCT: behaviour change technique
BCTTv1: behaviour change technique taxonomy version 1
CRSO: Cochrane Register of Studies Online
CTAGSR: Cochrane Tobacco Addiction Review Group Specialized Register
IC-SMOKE: identifying effective behavioural components of Intervention and Comparison group support provided in SMOKing cEssation interventions
RCT: randomized controlled trial
TAU: treatment-as-usual
