# Supplementary material for: Identifying effective behavioural components of Intervention and Comparison group support provided in SMOKing cEssation (IC-SMOKE) interventions: a systematic review protocol
Source: Syst Rev. 2016 May 4;5:77. doi: 10.1186/s13643-016-0253-1 (PMC4857384; doi:10.1186/s13643-016-0253-1)
Supplement: Additional file 1: — Prisma checklist. (DOCX 41 kb) [file 13643_2016_253_MOESM1_ESM.docx]

**PRISMA-P (Preferred Reporting Items for Systematic review and Meta-Analysis Protocols) 2015 checklist: recommended items to address in a systematic review protocol***

| Section and topic | Item No | Checklist item |
| --- | --- | --- |
| ADMINISTRATIVE INFORMATION | | |
| Title: |  |  |
| Identification | 1a | Identify the report as a protocol of a systematic review  *The protocol is identified as a systematic review in the article title: ‘Identifying effective behavioural components of Intervention and Comparison group support provided in SMOKing cEssation interventions (IC-SMOKE): A systematic review protocol.’* |
| Update | 1b | If the protocol is for an update of a previous systematic review, identify as such  *Not applicable.* |
| Registration | 2 | If registered, provide the name of the registry (such as PROSPERO) and registration number  *The protocol is registered at PROSPERO: CRD42015025251. This information is provided on the abstract page.* |
| Authors: |  |  |
| Contact | 3a | Provide name, institutional affiliation, e-mail address of all protocol authors; provide physical mailing address of corresponding author  *All names, institutional affiliations, email addresses of protocol authors and a physical mailing address for the corresponding author are provided on the title page of the manuscript.* |
| Contributions | 3b | Describe contributions of protocol authors and identify the guarantor of the review  *Contributions of protocol authors are specified under the header ‘Author contributions’ at the end of the manuscript.* |
| Amendments | 4 | If the protocol represents an amendment of a previously completed or published protocol, identify as such and list changes; otherwise, state plan for documenting important protocol amendments  *Not applicable.* |
| Support: |  |  |
| Sources | 5a | Indicate sources of financial or other support for the review |
| Sponsor | 5b | Provide name for the review funder and/or sponsor |
| Role of sponsor or funder | 5c | Describe roles of funder(s), sponsor(s), and/or institution(s), if any, in developing the protocol  *Information on the role of the funder is provided under the header ‘Funding’ at the end of the manuscript.* |
| INTRODUCTION | | |
| Rationale | 6 | Describe the rationale for the review in the context of what is already known  *The rationale of the review is provided in the Introduction.* |
| Objectives | 7 | Provide an explicit statement of the question(s) the review will address with reference to participants, interventions, comparators, and outcomes (PICO)  *General objectives and specific research questions are specified under the section ‘Objectives’ at the end of the introduction.* |
| METHODS | | |
| Eligibility criteria | 8 | Specify the study characteristics (such as PICO, study design, setting, time frame) and report characteristics (such as years considered, language, publication status) to be used as criteria for eligibility for the review  *Eligibility criteria are described under the header ‘Search and screening process’.* |
| Information sources | 9 | Describe all intended information sources (such as electronic databases, contact with study authors, trial registers or other grey literature sources) with planned dates of coverage  *Information sources are described in the method section under ‘Overview of the search strategy’, ‘Search and screening process’ and ‘data collection’.* |
| Search strategy | 10 | Present draft of search strategy to be used for at least one electronic database, including planned limits, such that it could be repeated  *The search strategy is specified under the headers ‘Overview of the search strategy’ and ‘Search and screening process’.* |
| Study records: |  |  |
| Data management | 11a | Describe the mechanism(s) that will be used to manage records and data throughout the review  *Records will be managed using Endnote and MS Excel as reported in the section ‘Search and screening process’* |
| Selection process | 11b | State the process that will be used for selecting studies (such as two independent reviewers) through each phase of the review (that is, screening, eligibility and inclusion in meta-analysis)  *The screening process is described under the header ‘Overview of the search strategy’, ‘Search and screening process’.* |
| Data collection process | 11c | Describe planned method of extracting data from reports (such as piloting forms, done independently, in duplicate), any processes for obtaining and confirming data from investigators  *Data collection methods are described under the header ‘Data collection’.* |
| Data items | 12 | List and define all variables for which data will be sought (such as PICO items, funding sources), any pre-planned data assumptions and simplifications  *Variables that will be extracted are specified under the section ‘Data collection’, under the sub-header ‘Data extraction’.* |
| Outcomes and prioritization | 13 | List and define all outcomes for which data will be sought, including prioritization of main and additional outcomes, with rationale  *Variables that will be extracted are specified under the section ‘Data collection’, under the sub-header ‘Data extraction’.* |
| Risk of bias in individual studies | 14 | Describe anticipated methods for assessing risk of bias of individual studies, including whether this will be done at the outcome or study level, or both; state how this information will be used in data synthesis  *As specified in the section ‘Data collection’, under the sub-header ‘Data extraction’ risk of bias will be assessed with the risk of bias Cochrane tool and with the recently developed RATIONALE table, which includes various items to assess risk of bias in social sciences research. Risk of bias will be included as a potential covariate in analyses of the effects of (clusters of) behaviour change techniques on smoking cessation rates, as specified in the section ‘Statistical analyses’* |
| Data synthesis | 15a | Describe criteria under which study data will be quantitatively synthesised  *This information is described at the top of the section 'Statistical analyses'* |
|  | 15b | If data are appropriate for quantitative synthesis, describe planned summary measures, methods of handling data and methods of combining data from studies, including any planned exploration of consistency (such as I^2^, Kendall’s τ)  *This information is provided in the section ‘Statistical analyses’.* |
|  | 15c | Describe any proposed additional analyses (such as sensitivity or subgroup analyses, meta-regression)  *This information is provided in the section ‘Statistical analyses’.* |
|  | 15d | If quantitative synthesis is not appropriate, describe the type of summary planned  *Not applicable.* |
| Meta-bias(es) | 16 | Specify any planned assessment of meta-bias(es) (such as publication bias across studies, selective reporting within studies)  Assessment of risk of publication bias is assessed, as reported in the section ‘Statistical analyses’ |
| Confidence in cumulative evidence | 17 | Describe how the strength of the body of evidence will be assessed (such as GRADE)  *The strength of the body of evidence will be assessed using GRADE as mentioned in the section ‘Dissemination of findings’* |

*** It is strongly recommended that this checklist be read in conjunction with the PRISMA-P Explanation and Elaboration (cite when available) for important clarification on the items. Amendments to a review protocol should be tracked and dated. The copyright for PRISMA-P (including checklist) is held by the PRISMA-P Group and is distributed under a Creative Commons Attribution Licence 4.0.**

*From: Shamseer L, Moher D, Clarke M, Ghersi D, Liberati A, Petticrew M, Shekelle P, Stewart L, PRISMA-P Group. Preferred reporting items for systematic review and meta-analysis protocols (PRISMA-P) 2015: elaboration and explanation. BMJ. 2015 Jan 2;349(jan02 1):g7647.*
